# Rotavirus Surveillance in Urban and Rural Areas of Niger, April 2010–March 2012

**DOI:** 10.3201/eid2004.131328

**Published:** 2014-04

**Authors:** Anne-Laure Page, Viviane Jusot, Abdoul-Aziz Mamaty, Lagare Adamou, Jérôme Kaplon, Pierre Pothier, Ali Djibo, Mahamane L. Manzo, Brahima Toure, Céline Langendorf, Jean-Marc Collard, Rebecca F. Grais

**Affiliations:** Epicentre, Paris, France (A.-L. Page, C. Langendorf, R.F. Grais); Epicentre, Niamey, Niger (V. Jusot, A.-A. Mamaty, B. Toure);; Centre de Recherche Médicale et Sanitaire (CERMES), Niamey (L. Adamou, J.-M. Collard);; University Hospital of Dijon, Dijon, France (J. Kaplon, P. Pothier);; Ministry of Health, Niamey (A. Djibo, M.L. Manzo);; Niamey University, Niamey (A. Djibo)

**Keywords:** rotavirus, genotyping, diarrheal diseases, Niger, developing countries, malnutrition, Africa, rotavirus strain G12P[8], surveillance, gastroenteritis, viruses

## Abstract

Knowledge of rotavirus epidemiology is necessary to make informed decisions about vaccine introduction and to evaluate vaccine impact. During April 2010–March 2012, rotavirus surveillance was conducted among 9,745 children <5 years of age in 14 hospitals/health centers in Niger, where rotavirus vaccine has not been introduced. Study participants had acute watery diarrhea and moderate to severe dehydration, and 20% of the children were enrolled in a nutrition program. Of the 9,745 children, 30.6% were rotavirus positive. Genotyping of a subset of positive samples showed a variety of genotypes during the first year, although G2P[4] predominated. G12 genotypes, including G12P[8], which has emerged as a predominant strain in western Africa, represented >80% of isolates during the second year. Hospitalization and death rates and severe dehydration among rotavirus case-patients did not differ during the 2 years. The emergence of G12P[8] warrants close attention to the characteristics of associated epidemics and possible prevention measures.

As the leading cause of severe gastroenteritis in children, rotavirus is responsible for ≈450,000 deaths each year among children <5 years of age, mainly in low-income countries (*1*,*2*). Two rotavirus vaccines that have been prequalified by the World Health Organization, Rotarix (GlaxoSmithKline Biologicals, Rixensart, Belgium) and Rotateq (Merck, Whitehouse Station, NJ, USA), have been introduced widely in high- and middle-income countries, where their effect on rotavirus-related hospital admissions and deaths has been demonstrated (*3*). These vaccines are being introduced in several low-income countries in Africa and Asia, where their efficacy is lower (*4*–*6*) and impact data are limited (*7*).

Four rotavirus genotypes were historically recognized as predominant: G1P[8], G2P[4], G3P[8], and G4P[8]. These genotypes represented 88% of all rotavirus strains worldwide, and genotype G1P[8] has been responsible for >70% of the rotavirus infections in North America, Europe, and Australia (*8*). Since 2000, the prevalence of G1 strains has been declining, and other genotypes, such as G9 and G12, have emerged (*9*). In Africa, G12 strains were first detected in southern Africa, mostly in association with P[6] (*10*–*12*), and G12P[8] recently emerged as a predominant strain in western Africa (*13*,*14*) and some regions of Spain (*15*), Argentina (*16*), and the United States (*17*).

Because questions remain about vaccine efficacy and impact in low-resource settings, countries planning to introduce rotavirus vaccine must have knowledge of rotavirus epidemiology and circulating genotypes to evaluate the potential effect of vaccine programs. Niger is one of 34 countries approved by GAVI Alliance for financial support, but rotavirus vaccine has not yet been introduced in the country. We present rotavirus surveillance data for children <5 years of age in 14 hospitals and health centers in urban and rural Niger.

## Patients and Methods

### Study Sites

Gastroenteritis surveillance was conducted in 2 urban areas (Niamey, the capital city of Niger, and Maradi, the administrative center of the Maradi region) and in 3 rural districts (Madarounfa, Aguié, and Guidan Roumdji) in Maradi region, which is located ≈500 km from Niamey. Results of a 2009 survey of health-seeking behavior showed that hospital-based surveillance would capture <10% of severe diarrhea cases (*18*); thus, to capture more cases, we included hospitals and health centers in the study. Surveillance was implemented in several stages, beginning in December 2009 at 3 health centers in Madarounfa and at the regional hospital in Maradi. In 2010, a total of 10 other sites were added to the surveillance: in January, 3 health centers in Aguié were included; in February, 4 health centers in Guidan Roumdji were included; and in April, the 3 main hospitals in Niamey were included. After 1 year of surveillance, the number of sites was reduced for organizational and budgetary reasons. The analysis presented here is restricted to a 24-month period, April 2010–March 2012.

### Study Population

Children 0–59 months of age were included in the study if they sought medical care at a study site, had watery diarrhea and signs of moderate or severe dehydration, and their parents or legal guardians accepted participation in the study. Children in Maradi region with reported bloody diarrhea were included during the second year, but they were excluded from this analysis.

Watery diarrhea was defined as >3 loose or liquid stools per day. Moderate and severe dehydration were defined on the basis of the child’s general state and on thirst and ability to drink, sunken eyes, and skin-pinch assessment, according to the Integrated Management of Childhood Illness guidelines (*19*). The Vesikari score was calculated at the medical consultation or hospital admission (*20*). Vesikiari scores >11 were considered as severe.

### Data Collection

A standardized questionnaire was used to obtain sociodemographic data, clinical signs and symptoms at the first medical visit, and outcomes. Enrolment of the child in a nutrition program was also recorded. If the child was transferred to another health facility, outcome information was obtained at the transfer site. If a child died at a health facility, the reported cause(s) of death, as assessed by the clinicians in charge, was recorded.

### Specimen Collection and Rotavirus Assay

Fecal specimens were collected into sterile plastic containers or by using a rectal swab. We performed the Vikia Rota-Adeno rapid test (bioMérieux, Marcy l'Etoile, France) on site, following the manufacturer’s recommendations.

Rotavirus-positive specimens and a sample of negative specimens were stored in a cool box and transported to the central laboratories in Maradi (every other day) and Niamey (every day), where they were aliquoted and stored at −20°C. At the central laboratories, the rapid test was repeated on ≈10% of the samples; good concordance was found with the original test results (κ 0.83). When discordant results were reported, training on use and interpretation of the rapid test was reinforced at the concerned study site(s).

### Genotyping

For the first year of surveillance, a sample size of 420 specimens for genotyping was calculated to estimate the most frequent genotype expected at a 50% level with ±5% precision. For each 3-month period during the first year (April 1, 2010–March 31, 2011), a stratified random selection was used to select 70 and 35 specimens from Maradi region and Niamey, respectively, from the list of rotavirus-positive samples; selection was made regardless of the health facility of origin. A descriptive sample of 150 specimens (100 from the Maradi region, 50 from Niamey) was used to follow genotype evolution during the second year of surveillance (April 1, 2011–March 31, 2012). These samples were randomly selected separately for Niamey and Maradi at the end of the second year.

Rotavirus genotyping was conducted at CERMES (Niamey), according to the EuroRotaNet method (www.eurorota.net/docs.php), with the exception of the G12 primer. For the specimens that could not be genotyped, VP6 gene amplification was performed (*21*); the VP4 and/or VP7 first-round reverse transcription PCR (RT-PCR) products were sequenced directly at the National Reference Center for Enteric Viruses (Dijon, France) by using the ABI PRISM Big Dye Terminator Cycle Sequencing Kit on a 3130XL DNA Genetic Analyzer (Applied Biosystems, Foster City, CA, USA). For the specific amplification of rotavirus G12 strains, a new set of primers (forward primer: G12Fcnr, 5′-GTTGTCGTCATACTGCCAT-3′, nt 169–187; reverse primer: G12Rcnr 5′-ATGAATTTTGGTACTGTATT-3′, nt 471–490) was designed on the basis of the VP7 coding sequences of G12 strains from Niger and other countries.

### Statistical Analysis

We used EpiData version 3.1 (EpiData, Odense, Denmark) for double data entry and Stata version 12.1 (College Station, TX, USA) for data analysis. We performed a weighted analysis by month and region of study inclusion to extrapolate the results of genotyping of a random subset of rotavirus-positive fecal samples to the population of rotavirus-positive patients.

### Ethical Considerations

Ethical approval was granted by the National Ethics Committee of Niger (reference no. 02/2009/CCNE) and the Comité de Protection des Personnes, Ile de France XI, Saint-Germain en Laye, France. Written informed consent was obtained from each participant’s parent or legal guardian; study participation was voluntary.

## Results

### Characteristics of the Study Population

In total, 12,355 children with diarrhea and dehydration sought care at the study sites during April 2010–March 2012, of whom 2,038 (16%) were not included in the study for the following reasons: seeking care outside of the study hours, 1,183 (9.6%) children; reporting bloody diarrhea, 837 (6.8%) children; and refusing study participation, 18 (0.1%) children. We further excluded 270 children with bloody diarrhea, 35 children with who were not tested for rotavirus, 127 children with a delay of >3 days between hospital admission and testing, 134 children who sought care >14 days after onset of diarrhea, and 4 children without clinical signs of dehydration. Thus, we included 9,747 children in the analysis. Although sociodemographic characteristics for the children were similar between districts, other characteristics (e.g., degree of dehydration, receipt of intravenous treatment, and percentage hospitalized) differed because of differences in the level of care between study sites (i.e., hospital vs. health centers) (Table 1).

**Table 1:** Sociodemographic and clinical and treatment characteristics of children in a rotavirus surveillance study in Niamey and Maradi region, Niger, April 2010–March 2012*

Characteristic	Total, N = 9,747	Children, by location				
		Niamey, n = 1,196	Maradi region, district			
			Maradi, n = 962	Madarounfa, n = 2,965	Aguie, n = 748	Guidan Roumdji, n = 3,876
Sex						
F	4,329 (44.4)	481 (40.2)	442 (46.0)	1,401 (47.3)	309 (41.3)	1,696 (43.8)
M	5,416 (55.6)	715 (59.8)	518 (54.0)	1,564(52.8)	439 (58.7)	2,180 (56.2)
Age, mo, median (IQR)	9 (7–12)	9 (6–12)	9 (6–14)	9 (7–12)	8 (6–11)	10 (7–13)
Type of sample collected						
Stool	6,765 (69.5)	678 (56.7)	749 (77.9)	2,224 (75.1)	665 (88.9)	2,449 (63.3)
Rectal swab	2,974 (30.5)	518 (43.3)	213 (22.1)	739 (24.9)	83 (11.1)	1,421 (36.7)
Clinical signs/symptoms						
Severe dehydration	1,976 (20.3)	724 (60.5)	382 (39.8)	158 (5.3)	175 (23.4)	537 (13.9)
Fever	2,360 (24.2)	410 (34.3)	290 (30.2)	593 (20.0)	251 (33.6)	816 (21.1)
Vomiting	6,499 (66.8)	818 (68.6)	353 (36.7)	2,146 (72.5)	545 (72.9)	2,637 (68.3)
Severe Vesikari score rating	7,156 (73.5)	963 (80.6)	631 (65.7)	2,227 (75.2)	563 (75.5)	2,772 (71.7)
IV treatment received	1,392 (14.3)	599 (50.1)	577 (60.0)	56 (1.9)	29 (3.9)	131 (3.4)
Hospitalized	2,529 (26.0)	917 (76.7)	924 (96.2)	153 (5.2)	57 (7.6)	478 (12.3)
Enrolled in nutrition program	2,046 (21.0)	396 (33.2)	539 (56.0)	314 (11.0)	93 (12.4)	704 (18.2)
Died	255 (2.6)	119 (10.0)	48 (5.0)	50 (1.7)	16 (2.1)	22 (0.6)
*Data are no. (%) unless otherwise indicated. IQR, interquartile range; IV, intravenous.						

### Proportion of Rotavirus-associated Gastroenteritis

Overall, 2,982 (30.6%) children were positive for rotavirus. The percentage of positive study participants varied substantially across sites, especially during the first year (Table 2). Overall and after stratifying by type of site (hospital vs. health center) and for children included in a nutrition program, the percentage of rotavirus-positive cases was lower among hospitalized patients, those with severe dehydration, and those included in a nutrition program (Table 3).

**Table 2:** Number of rotavirus-positive study participants identified during a 2-year surveillance study in Niamey and Maradi region, Niger, April 2010–March 2012*

Variable	April 2010–March 2011			April 2011–March 2012	
	No. positive/no. total (%)	95% CI		No. positive/no. total (%)	95% CI
Study site					
All areas	1,714/5,845 (29.3)	28.2–30.5		1,268/3,902 (32.5)	31.0–34.0
Niamey†	180/796 (22.6)	19.7–25.5		148/478 (32.3)	27.7–36.8
Maradi region, district					
Maradi	95/484 (19.6)	16.1–23.2		105/478 (22.0)	18.2–25.7
Madarounfa	611/1,711 (35.7)	33.4–38.0		478/1,254 (38.1)	35.4–40.8
Aguie	252/748 (33.7)	30.3–37.1		NA	NA
Guidan Roumdji	576/2,106 (27.4)	25.5–29.3		556/1,770 (31.4)	29.2–33.6
Patients with stool samples tested	1,312/4,074 (32.2)	30.8–33.6		941/2,691 (34.9)	33.2–36.8
Niamey†	110/409 (26.9)	22.6–31.2		100/269 (37.1)	31.4–42.9
Maradi region, district					
Maradi	80/349 (22.9)	18.5–27.3		95/400 (23.8)	19.6–27.9
Madarounfa	485/1,293 (37.5)	34.9–40.2		364/931 (39.1)	36.0–42.2
Aguie	231/665 (34.7)	31.1–38.4		NA	NA
Guidan Roumdji	406/1,358 (29.9)	27.5–32.3		382/1,091 (35.0)	32.2–37.9

*NA, not applicable because surveillance was interrupted in all study sites in the district of Aguie at the end of the first year. †Surveillance was interrupted in 1 hospital in Niamey at the end of the first year.

**Table 3:** Number and percentage of rotavirus-positive study participants in a surveillance study in urban and rural areas of Niger, April 2010–March 2012

Variable	Urban and rural, N = 9,747			Urban, n = 2,158*			Rural, n = 7,589†	
	No. positive/ no. total (%)	95% CI		No. positive/ no. total (%)	95% CI		No. positive/ no. total (%)	95% CI
All patients	2,982 (30.6)	29.7–31.5		509 (23.6)	21.8–25.4		2,473 (32.6)	31.5–33.6
Hospitalization status								
Hospitalized	628/2,529 (24.8)	23.1–26.5		428/1,841 (23.2)	21.3–25.2		200/688 (29.1)	25.7–32.5
Not hospitalized	2,353/7,217 (32.6)	31.5–33.7		80/316 (25.3)	20.5–30.1		2,273/6,901 (32.9)	31.8–34.0
Dehydration status								
Severe	491/1,976 (24.8)	22.9–26.8		230/1,106 (20.8)	18.4–23.2		261/870 (30.0)	27.0–33.0
Moderate	2,490/7,770 (32.0)	31.0–33.1		278/1,051 (26.5)	23.8–29.1		2,212/6,719 (32.9)	31.8–34.0
Nutrition program status								
Enrolled	415/2,046 (20.3)	18.5–22.0		149/935 (15.9)	13.6–18.3		266/1,111 (23.9)	21.4–26.5
Not enrolled	2,564/7,687 (33.4)	32.3–34.4		359/1,221 (29.4)	26.8–32.0		2,205/6,466 (34.1)	32.9–35.3
Patients with stool sample tested	2,253/6,765 (33.3)	32.2–34.4		385/1,427 (27.0)	24.7–29.3		1,868/5,338 (35.0)	33.7–36.3
Hospitalization status								
Hospitalized	456/1,669 (27.3)	25.2–29.5		336/1,274 (26.4)	24.0–28.8		120/395 (30.4)	25.8–34.9
Not hospitalized	1,796/5,095 (35.3)	33.9–36.6		48/152 (31.6)	24.2–39.0		1,748/4,943 (35.3)	34.0–36.7
Dehydration status								
Severe	356/1,269 (28.1)	25.6–30.5		171/713 (24.0)	20.8–27.1		185/556 (33.3)	29.4–37.2
Moderate	1,896/5,495 (34.5)	33.2–35.8		213/713 (29.9)	26.5–33.2		1,683/4,782 (35.2)	33.8–36.5
Nutrition program status								
Enrolled	265/1,235 (21.5)	19.2–23.7		111/613 (18.1)	15.1–21.2		154/622 (24.8)	21.4–28.2
Not enrolled	1,985/5,521 (36.0)	34.7–37.2		273/813 (33.6)	30.3–21.2		1,712/4,708 (36.4)	35.0–37.7

*Hospitals in Niamey and Maradi. †Health centers in Madarounfa, Guidan Roumdji, and Aguie districts.

The percentage of rotavirus-positive specimens was lower among children from whom rectal swab samples (24.4%, 725/2,974) rather than stools samples (33.3%, 2,253/6,765) were obtained (p<0.001). This variation could not explain the difference in the percentage of positive cases by site and by clinical characteristics because these differences remained when the analysis was restricted to patients from whom stool samples (not rectal swab samples) were obtained (Tables 2, 3).

Children <1 year of age represented 67.4% of all diarrhea cases and 79.1% of rotavirus-positive cases (Technical Appendix Figure 1). More specifically, children <6 months of age accounted for 16.9% of all rotavirus-positive cases, and those 6–11 months of age accounted for 62.2%.

Rotavirus infections occurred year-round, and there was a consistent peak in October–November in Maradi region and in November–December in Niamey (Technical Appendix Figure 2). The 2011 peaks in March and April in Guidan Roumdji and Madarounfa, respectively, were not observed in 2010, and only a small increase was seen in the first trimester of 2012.

### Clinical Signs and Severity

The percentage of children with vomiting was consistently higher among rotavirus-infected than noninfected children, but the percentage with severe dehydration was lower (Table 4). The median Vesikari score was almost 1 point higher in children with rotavirus (Table 4).

**Table 4:** Age and clinical characteristics of study participants in a 2-year rotavirus surveillance study in urban and rural areas of Niger, April 2010–March 2012*

Patient variable	All participants, April 2010–March 2012, N = 9,747				RV-positive participants, n = 2,982		
	RV negative	RV positive	p value		First year†	Second year‡	p value
Age, mo, mean (± SD)	11.8 (6.9)	8.8 (4.4)	0.0001		9.1 (4.2)	8.4 (3.9)	0.0001
No. stools in 24-h, mean (± SD)	6.1 (1.7)	6.4 (1.8)	0.0001		6.4 (1.9)	6.4 (1.7)	0.76
Vomiting present, % (95% CI)	60.0 (58.9–61.2)	82.3 (80.9–83.7)	<0.001		81.8 (80.0–83.6)	83.0 (81.0–85.1)	0.37
No. vomiting episodes in 24-h, mean (± SD)	3.8 (1.9)	4.5 (2.8)	0.0001		4.6 (2.2)	4.4 (3.5)	0.012
Severe dehydration present, % (95% CI)	22.0 (21.0–22.9)	16.5 (15.1–17.8)	<0.001		17.2 (15.4–18.9)	15.5 (13.6–17.5)	0.23
Fever present, % (95% CI)	24.4 (23.4–25.5)	23.7 (22.1–25.2)	0.2		25.9 (23.9–28.0)	20.6 (18.4–22.8)	0.001
Vesikari score, mean (± SD)	12.1 (2.7)	12.9 (2.2)	0.0001		13.0 (2.2)	12.9 (2.1)	0.057
Hospitalized, % (95% CI)	28.3 (27.2–29.3)	20.8 (19.4–22.2)	<0.001		22.9 (20.9–24.9)	18.5 (16.4–20.7)	0.003
Died, % (95% CI)	3.3 (2.8–3.7)	1.1 (0.8–1.5)	<0.001		1.3 (0.8–1.9)	0.9 (0.4–1.4)	0.2

*RV, rotavirus. †April 2010–March 2011. ‡April 2011–March 2012. Vesikari score >11 indicates severe diarrhea.

Of the 9,747 study participants, 255 (2.6%) died in health facilities; 34 (13.3%; 95% CI 9.1%–17.5%) of those who died were rotavirus positive (Table 4). The most frequently cited causes of death among all patients who died were diarrhea or dehydration (124 [48.6%] patients; 95% CI 42.4%–54.8%) and malnutrition (109 [42.7%] patients; 95% CI 36.6%–48.9%). Of the 124 study participants with diarrhea and/or dehydration cited as a cause of death, 23 (18.9%) were positive for rotavirus.

### Rotavirus Genotypes

A total of 570 fecal samples that were positive for rotavirus by the rapid test were randomly selected for genotyping. Two of these specimens were lost, 3 were excluded because the delay between patient hospital admission and specimen collection was >3 days, and 2 were excluded because the patients had bloody diarrhea. Of the remaining 563 samples, 58 (10.3%) were rotavirus negative by RT-PCR and 56 were excluded because they were collected before April 2010; the latter group of samples comprised 15 G2P[6], 14 G1P[8], 12 G2P[4], 3 G12P[8], 10 mixed infection, and 2 partially typed samples.

A variety of G and P combinations were detected during the study, but G12P[8] and G2P[4] were the most prevalent rotavirus strains (Table 5). The seasonal distribution of these 2 genotypes extrapolated to all rotavirus-positive patients showed a clear shift to predominance of G12P[8] during the second year (Figure).

**Table 5:** Weighted analysis of rotavirus genotypes identified during a 2-year surveillance study in Niamey and Maradi region, Niger, April 2010–March 2012

Genotype and G- and P-type	April 2010–March 2011						April 2011–March 2012					
	Niamey, n = 121			Maradi region, n = 194			Niamey, n = 49			Maradi region, n = 85		
	No. (%)	95% CI		No. (%)	95% CI		No. (%)	95% CI		No. (%)		95% CI
Genotype												
G1P[8]	6 (4.8)	2.1–10.5		3 (1.8)	0.6–5.7		1 (1.8)	0.2–12.4		4 (3.8)		1.3–10.6
G2P[4]	30 (25.1)	18.0–33.9		137 (60.5)	51.6–68.8		0	–		0		–
G2P[6]	9 (6.2)	3.2–11.6		5 (3.7)	9.0–1.6		0	0		2 (2.7)		0.6–10.3
G6P[6]	12 (9.4)	5.4–16.1		0	–		1 (1.3)	0.2–9.2		0		–
G9P[8]	8 (5.9)	2.9–11.6		7 (5.9)	2.8–11.9		0	–		1 (1.3)		0.2–8.9
G12P[8]	36 (31.4)	23.4–40.5		19 (10.9)	6.6–17.5		43 (89.2)	76.3–95.5		56 (64.1)		52.4–74.4
Others*	15 (13.2)	8.0–21.0		13 (10.0)	5.4–17.8		2 (2.7)	0.6–11.4		9 (12.3)		6.1–23.3
Mixed	5 (4.0)	1.6–9.6		10 (7.1)	3.5–13.7		2 (5.1)	1.2–19.3		13 (15.8)		9.1–26.1
G-type†												
G1	10 (8.1)	4.3–14.7		4 (2.7)	1.0–7.1		2 (4.8)	1.1–18.7		4 (3.7)		1.3–10.6
G2	51 (41.9)	33.2–51.1		148 (66.5)	57.5–74.4		0	–		6 (7.9)		3.5–16.7
G3	3 (2.7)	0.8–8.5		17 (16.0)	10.0–24.7		0	–		5 (6.0)		2.4–14.3
G6	13 (10.3)	6.0–17.2		1 (0.1)	0.0–1.0		2 (3.0)	0.7–12.3		0		–
G9	10 (7.2)	3.8–13.1		13 (11.1)	6.4–18.5		1 (2.1)	0.3–14.2		9 (10.0)		5.0–18.7
G12	36 (31.4)	23.4–40.5		20 (11.1)	6.8–17.6		46 (95.2)	85.3–98.5		71 (82.8)		72.3–89.9
P-type†												
P[4]	32 (27.0)	19.7–35.9		145 (67.8)	59.1–75.4		0	–		0		–
P[6]	27 (20.1)	13.9–28.0		14 (9.7)	5.6–16.2		2 (2.2)	0.5–9.0		13 (17.6)		10.1–28.9
P[8]	63 (53.7)	44.6–62.6		38 (23.6)	16.9–31.9		47 (97.8)	91.0–99.5		72 (83.7)	72.9–90.7	

*Including G1P[6] (2), G2P[8] (9), G3P[4] (5), G3P[6] (7), G3P[8] (2), G6P[8] (3), and G12P[6] (7). †Mixed infections counted in each G- or P-type found, potentially leading to a total number greater than N.

**Figure:**
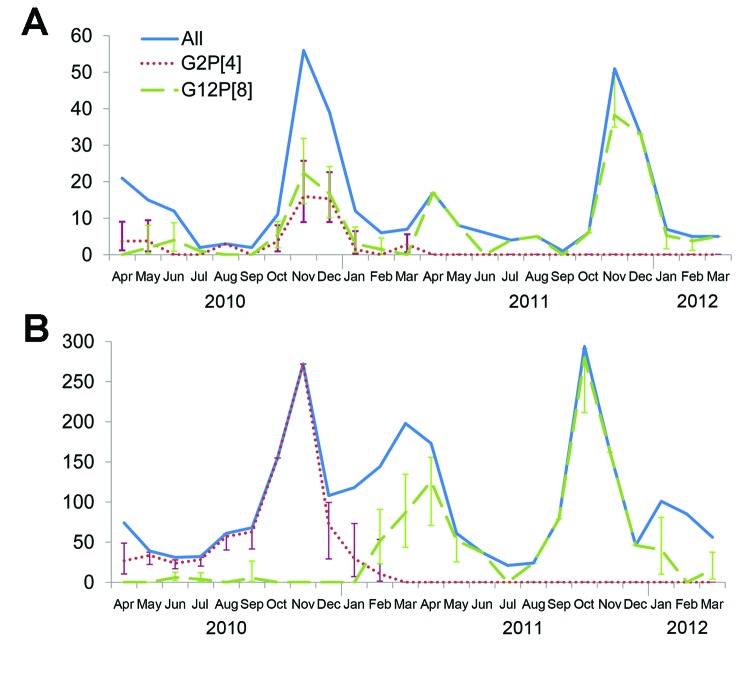
Number of rotavirus cases and extrapolated number of the 2 most frequent genotypes, G2P[4] and G12P[8], identified each month during a 2-year surveillance study in urban and rural areas of Niger, April 2010–March 2012. A) Cases in Niamey, the capital of Niger. B) Cases in Maradi region. Vertical bars indicate CIs.

### Comparison of First- and Second-Year Characteristics

Because rotavirus diarrhea was due to a variety of genotypes during the first year and primarily to G12 genotype during the second year, we compared characteristics of rotavirus cases for the 2 years to detect differences that could be linked to the G12 genotype. Although the percentage of rotavirus infections among study participants was substantially higher during the second year, the total number of rotavirus cases, and thus incidence of infection, was not higher in districts where the number of surveillance sites did not change during the 2 years (Table 2). Rotavirus-infected patients during the second year were significantly younger than those during the first year, but clinical characteristics for patients were similar during the 2 years, except for a lower percentage of patients with fever and a lower percentage of patient hospitalizations during the second year (Table 4).

## Discussion

The results of this large study on the epidemiology of rotavirus in urban and rural settings in Niger confirm the high morbidity rate for rotavirus in this area, particularly among children <1 year of age and during the dry and cool season. This 2-year study captured the emergence of G12P[8] strains, which are emerging in several areas of the world (*9*,*13*–*16*).

The emergence of G12P[8] warrants close attention to the characteristics of associated epidemics and possible prevention measures. Although only available for Rotarix vaccine, the first data on the efficacy of vaccine against rotavirus G12 strains in the clinical trials in Africa suggest that the vaccine provides heterotypic protection, although confidence intervals are wide (*22*). The lower mean age of rotavirus-infected children during the G12P[8] season, compared with the mean age during the previous year, emphasizes the need for early vaccination. A lower protection provided by maternal antibodies acquired transplacentally or through breast-feeding might partly explain the lower mean age of infection during the second year. Other characteristics of the rotavirus cases during the second year of the study suggested that G12P[8]-associated diarrhea might be clinically slightly less severe than diarrhea caused by other strains. Only 1 previous study compared characteristics of a G12 epidemic with characteristics of previous epidemics in which G1 or G9 strains were dominant, and no difference in mean age and hospitalization rates was found (*15*).

In the context of our study, rotavirus infection was not associated with severity criteria, except for the Vesikari score. In particular, severe dehydration, hospitalizations, and deaths were less frequent among children with than without rotavirus. One factor that might explain this result is the high prevalence of acute malnutrition; this factor was associated with a higher proportion of study participants who were hospitalized or who had severe dehydration but with a lower proportion of study participants with rotavirus infection. The lower percentage of rotavirus cases among children who were included in nutrition programs does not imply a lower incidence of rotavirus among these children. A study in South Africa reported that the percentage of rotavirus cases among HIV-infected children with diarrhea was lower than the percentage among non–HIV-infected children with diarrhea; however, when the overall incidence of acute gastroenteritis was taken into account, the incidence of rotavirus in HIV-infected children was 2.3-fold higher than in non–HIV-infected children (*23*). More precise data on the incidence and natural history of rotavirus infection and on vaccine efficacy in children with severe acute malnutrition are needed. Diarrhea-associated mortality data are also needed because the direct extrapolation of the percentage of rotavirus-associated diarrhea cases to diarrhea-associated deaths may not be relevant, particularly in contexts with high levels of malnutrition (*24*).

Our study has several limitations. First, malnutrition status was monitored only through the study participant inclusion in a nutrition program. In addition, children with severe acute malnutrition can show signs and symptoms similar to those of dehydration. However, in this study, the duration of diarrhea, the number of watery stools, and the Vesikari score were similar in children with or without malnutrition (data not shown), suggesting that dehydration was truly associated with diarrhea. Second, the rapid test used in this study is not ideal, as suggested by the fact that 10.3% of the rotavirus-positive stool samples selected for genotyping were negative by RT-PCR. The rapid test was chosen for practical reasons and for its good performance in initial evaluations (*25*,*26*), and the test had similar sensitivity but lower specificity than the Premier Rotaclone immunoassay (Meridian Bioscience, Inc., Cincinnati, Ohio, USA) in a post hoc evaluation (A.-L. Page, unpub. data). The lower test specificity might have led to a slightly higher estimate than if an immunoassay had been used. The last limitation is that the sensitivity of rotavirus detection was lower among patients for whom rectal swab samples rather than stool samples were used for testing; the difference could reflect the lower sensitivity of rectal swab sample testing. This lower sensitivity might have led to underestimating the percentage of rotavirus cases by ≈3% overall and by a slightly higher percent in Niamey and Guidan Roumdji, where the proportion of patients tested by using rectal swab samples was higher than in other study areas.

Rotavirus is a major cause of diarrhea with dehydration in Niger, but it is not associated with severity criteria (e.g., hospitalization, severe dehydration, and death), probably because of the large number of children with malnutrition. The emergence of G12P[8] in Niger and other areas of Africa indicates the complex natural evolution of rotavirus strains, even in the absence of any external pressure (e.g., vaccination), and the need for continuous surveillance. Better data on the efficacy and effect of existing and new rotavirus vaccines against currently circulating strains in countries with a high prevalence of malnutrition are crucially needed before the nationwide introduction of vaccine in these countries.

Technical Appendix Age distribution of rotavirus-positive and -negative children and number of rotavirus cases per month in Niamey and Maradi region, Niger, April 2010–March 2012.
