# Supplementary material for: Rotavirus Surveillance in Urban and Rural Areas of Niger, April 2010–March 2012
Source: Emerg Infect Dis. 2014 Apr;20(4):573–80. doi: 10.3201/eid2004.131328 (PMC3966376; doi:10.3201/eid2004.131328)
Supplement: Technical Appendix — Age distribution of rotavirus-positive and -negative children and number of rotavirus cases per month in Niamey and Maradi region, Niger, April 2010–March 2012. [file 13-1328-Techapp-s1.pdf]

Article DOI: http://dx.doi.org/10.3201/eid2004.131328

Rotavirus Surveillance in Urban and Rural Areas of Niger, April 2010–March 2012

Technical Appendix

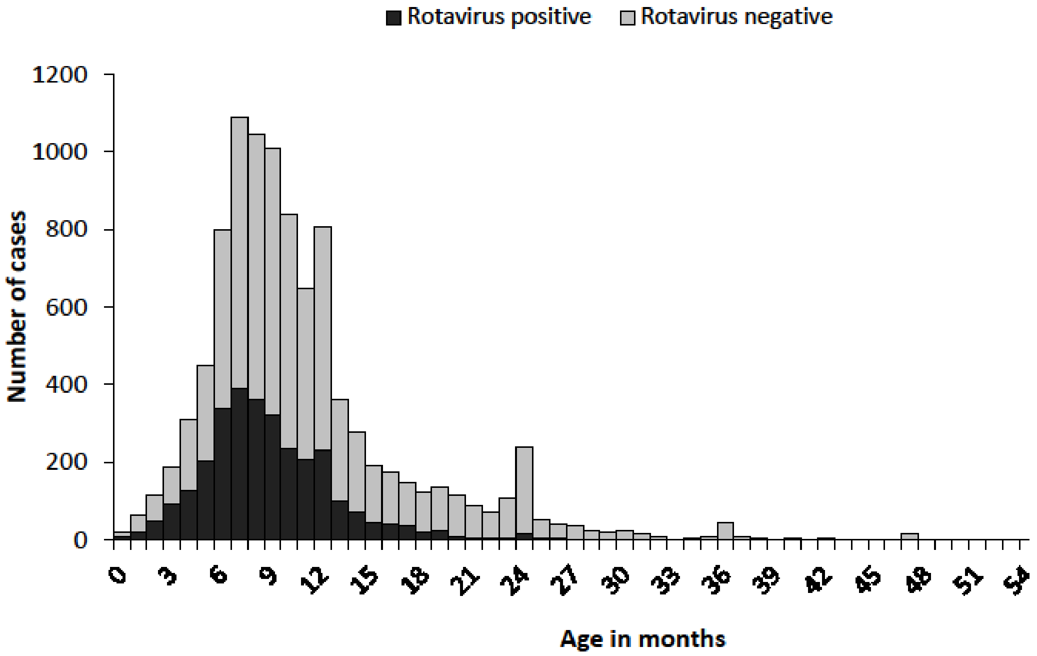

Technical Appendix Figure 1. Age distribution of rotavirus-positive and rotavirus-negative children in Niamey and Maradi region, Niger, April 2010–March 2012.

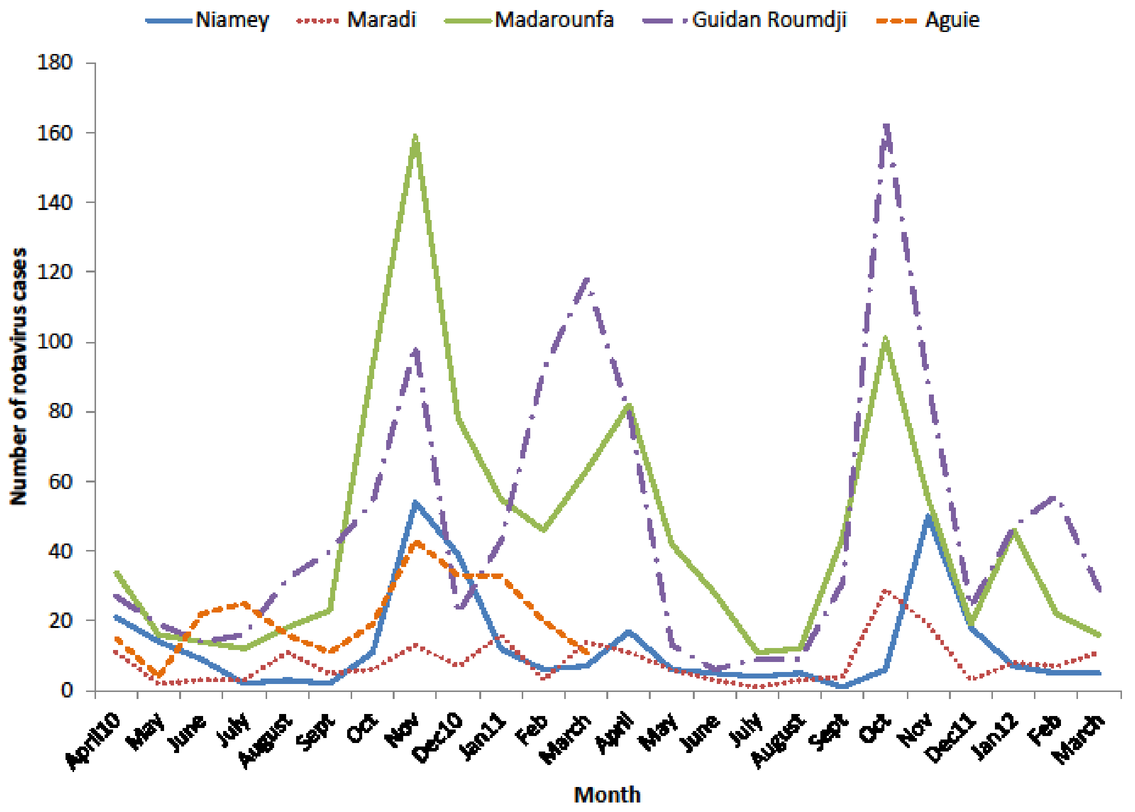

Technical Appendix Figure 2. Number of rotavirus cases per month in Niamey and Maradi region, Niger, April 2010–March 2012.
